# AGING IN COMMUNITY: LESSONS LEARNED IN THE FIRST YEAR OF DEVELOPING A GRASSROOTS, RURAL PROGRAM

**DOI:** 10.1093/geroni/igac059.1989

**Published:** 2022-12-20

**Authors:** Emily Kinkade, Melisa Hajdar, Heather Fuller

**Affiliations:** North Dakota State University, Fargo, North Dakota, United States; North Dakota State University, Fargo, North Dakota, United States; North Dakota State University, Fargo, North Dakota, United States

## Abstract

The North Dakota Aging in Community project has an overarching goal of creating programs that improve the quality of life of rural older adults by increasing community-level support to help them age in place in two rural communities. The progress of program development and implementation was assessed across the project’s first year through program data and steering committee surveys. Program data, including steering committee meeting minutes and monthly activity reports completed by program staff, were qualitatively analyzed for successes, challenges, and lessons learned by finding common themes within these data. Successes included the use of an apprenticeship/mentorship model utilizing consultants from similar grassroots, rural organizations, recruitment of an optimistic, enthusiastic, and collaborative steering committee, and employing patience to develop community-led efforts based on unique community needs. Challenges included hiring qualified staff in rural communities, limitations in infrastructure opportunities, and need for creative marketing strategies. Survey results aligned with these conclusions, yet demonstrated the strengths and challenges related to incorporating the ideas and needs of diverse stakeholders. These findings highlight the unique challenges in developing programs to support rural aging-in-place, yet also highlight unique strategies that leverage rural strengths. Based on the findings of this first-year evaluation, future directions include conducting needs assessments to identify new avenues for program development, ensuring local program ownership through continued development of local steering committees, and continuing the mentorship/apprenticeship model while creating strategies to successfully foster independence. By sharing lessons learned, this program may serve as a replicable model to foster rural aging-in-place.

